# The Unusual Cosubstrate Specificity of NQO2: Conservation Throughout the Amniotes and Implications for Cellular Function

**DOI:** 10.3389/fphar.2022.838500

**Published:** 2022-04-20

**Authors:** Faiza Islam, Kevin K. Leung, Matthew D. Walker, Shahed Al Massri, Brian H. Shilton

**Affiliations:** ^1^ Department of Biochemistry, University of Western Ontario, London, ON, Canada; ^2^ Department of Pharmaceutical Chemistry, University of California, San Francisco, San Francisco, CA, United States

**Keywords:** quinone reductase, pseudoenzyme, evolution, amniotes, nicotinamide cofactor, NAD(P)H

## Abstract

Human Quinone Reductase 2 (NQO2) is a pharmacological target and has appeared in numerous screening efforts as an off-target interactor with kinase-targeted drugs. However the cellular functions of NQO2 are not known. To gain insight into the potential cellular functions of NQO2, we have carried out a detailed evolutionary analysis. One of the most striking characteristics of NQO2 is that it uses conventional dihydronicotinamide cosubstrates, NADH and NADPH, extremely inefficiently, raising questions about an enzymatic function in cells. To characterize the ability of NQO2 to serve as an enzyme, the NQO2 gene was disrupted in HCT116 cells. These NQO2 knockouts along with the parental cells were used to demonstrate that cellular NQO2 is unable to catalyze the activation of the DNA cross-linking reagent, CB1954, without the addition of exogenous dihydronicotinamide riboside (NRH). To find whether the unusual cosubstrate specificity of NQO2 has been conserved in the amniotes, recombinant NQO2 from a reptile, *Alligator mississippiensis*, and a bird, *Anas platyrhynchos*, were cloned, purified, and their catalytic activity characterized. Like the mammalian enzymes, the reptile and bird NQO2 were efficient catalysts with the small and synthetic cosubstrate *N*-benzyl-1,4-dihydronicotinamide but were inefficient in their use of NADH and NADPH. Therefore, the unusual cosubstrate preference of NQO2 appears to be conserved throughout the amniotes; however, we found that NQO2 is not well-conserved in the amphibians. A phylogenetic analysis indicates that NQO1 and NQO2 diverged at the time, approximately 450 MYA, when tetrapods were beginning to evolve.

## Introduction

Human Quinone Reductase 2 (NQO2) is an unusual enzyme. On the one hand, it is very closely related to NQO1, which functions in the reduction of quinones and other electrophiles. However, NQO1 and other members of the flavodoxin family use the cosubstrates NADH and/or NADPH for reduction of the flavin, which then transfers the electrons to the quinone or another electrophile. NQO2, in contrast, is reduced by NAD(P)H very inefficiently to the degree that its catalytic activity is very low and difficult to measure using NAD(P)H as the reducing cosubstrate. The unusual cosubstrate requirements for NQO2 were noted when it was originally purified and characterized 50 years ago in an excellent manuscript by Williams-Ashman and colleagues (Liao et al., 1962). In this manuscript, the group observed a menadione reduction activity that was dependent on dihydronicotinamide riboside (NRH) rather than NAD(P)H, and was present in extracts from a variety of tissues. By tracking this NRH-dependent activity, they were able to purify NQO2 from bovine kidneys (the tissue with the greatest NRH-dependent menadione reduction capability). The purified enzyme, now known as NQO2, had no significant activity with NAD(P)H as cosubstrate, but was active with NRH (Liao et al., 1962).

NQO2 is actually able to use a variety of small reducing dihydronicotinamide cosubstrates efficiently, many of which are synthetic and not present in living systems (Liao et al., 1962; Knox et al., 2000; Makarov et al., 2021). Of these, a potential *cellular* cosubstrate is NRH, which can be produced through the degradation of NADH. In fact, when NQO2 was originally characterized, it was suggested that its function was to scavenge cellular NRH to produce NR that could be “further degraded enzymatically” (Liao et al., 1962). Recent studies on nicotinamide metabolism confirm that NRH is present in cells as a degradation product of NAD(P)H, and can be used in a salvage pathway to produce NAD^+^ (Yang et al., 2019, 2020). The primary route in this pathway involves the phosphorylation of NRH by adenosine kinase to dihydronicotinamide mononucleotide (NMNH) and then conversion to NAD^+^; there is no indication that NQO2 is a required part of this pathway (Yang et al., 2020). Similarly, NR is known to participate in salvage pathways to regenerate NAD^+^ (Bieganowski and Brenner, 2004). Currently there are no enzymes or mechanisms known that could reduce nicotinamide riboside (NR) to NRH, and NQO2 appears to be the only enzyme with a specificity for NRH over NAD(P)H, and so the redox cycling that is central to the function of NAD(P)H as carriers of reducing equivalents does not seem to apply in the case of NR and NRH. We continue to discover new functional aspects of nicotinamide and dihydronicotinamide nucleotide metabolism, and it is conceivable that NRH may serve as a major source of reducing equivalents under specific metabolic conditions where its recycling to NAD^+^ is blocked. However, our current state of knowledge regarding cellular NR and NRH indicates that they function primarily as intermediates in the synthesis and salvage pathways for NAD^+^. On this basis, if the role of NQO2 is to catalyze the reduction of electrophiles, it is not clear why NQO2 would evolve to use NRH as a cosubstrate rather than the conventional, and typically more abundant, dihydronicotinamide cosubstrates NADH and NADPH.

A second possibility for the unusual cosubstrate specificity of NQO2 is that it has evolved as a pseudo enzyme, a molecule that has shed the ability for efficient catalysis but retained and/or evolved a cellular function that is not dependent on a high catalytic efficiency. The most well-studied group of pseudo enzymes are the pseudo kinases (Kwon et al., 2019). Pseudo kinases contain significant sequence and structural similarity to kinases but have mutations in important active site residues coupled with little or no capacity for substrate phosphorylation. Nevertheless, the pseudo kinases have important functions in signal transduction pathways due to their interactions with active kinases and other molecules.

Flavin-linked enzymes are known to be integral players in the cellular responses to light and changes in cellular metabolites and redox potential. Examples of flavin redox switches are NifL, which regulates transcription of genes involved in nitrogen fixation; pyruvate oxidase, which resides in the cytosol when oxidized, but binds to the membrane when reduced; and the proline utilization A (PutA) enzyme, which has enzymatic functions, can act as a transcriptional regulator, and functions as a sensor of cellular metabolism by responding to proline levels (Becker et al., 2011). In the case of NQO2, we have observed that upon reduction and chloroquine binding, the enzyme undergoes a small but detectable conformational change, consistent with a potential redox switch function (Leung and Shilton, 2013). Along these lines, other flavodoxin-like reductases such as yeast Lot6p and NQO1 have been proposed to act as flavin-redox switches (Sollner et al., 2009a, 2009b; Siegel et al., 2018; Ross and Siegel, 2021). Furthermore, Boutin and co-workers have shown that NQO2 is able to generate reactive oxygen species, which may function in cellular signaling (Reybier et al., 2011; Cassagnes et al., 2015, 2018). On the basis of the very poor ability of NQO2 to use NAD(P)H, and the precedents for flavin-linked enzymes to play signaling and regulatory roles, we hypothesize that NQO2 may have non-enzymatic functions in redox sensing and/or cellular signal transduction.

To further explore the cosubstrate specificity of NQO2, we used genome editing approaches to knock out NQO2 from HCT116 cells, and show that the enzymatic activity of NQO2 in cells is entirely dependent on exogenous addition of small nicotinamide cosubstrates. To analyze its evolution, NQO2 enzymes from a reptile and bird were expressed, purified, and shown to have the same inability as mammalian NQO2 to use NAD(P)H efficiently. Further investigation into extant sequenced genomes indicates that NQO2 appeared in the early evolution of the amniotes. We expect that a more detailed analysis of the evolution of NQO1 and NQO2 will illuminate the changes in NQO2 that are responsible for its very inefficient use of NAD(P)H as a reducing cosubstrate.

## Materials and Methods

### Chemicals and Media

Nicotinamide riboside (NR) was purchased from High Performance Nutrition (Irving, CA) and dihydronicotinamide riboside (NRH) was prepared according to the previously described protocol (Knox et al., 2000). 1-Benzyl-1,4-dihydro-3-pyridinecarboxamide (BNAH) was purchased from TCI Chemicals.

### Generation of Human Quinone Reductase 2 Knockouts

**FIGURE 1:**
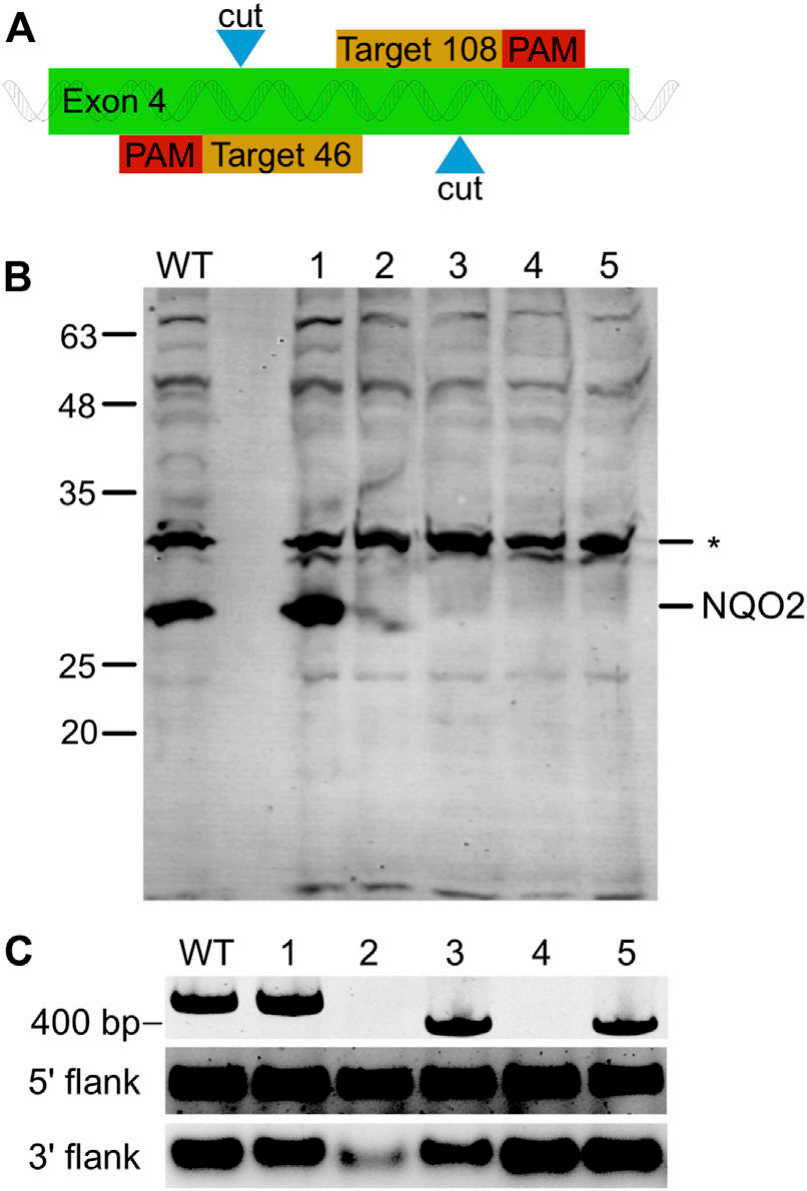
NQO2 Gene Knock-Out in HCT116 using CRISPR/Cas9 Nickase. **(A)** Illustration of the knockout strategy. The paired guide RNA was designed to generate a single strand nick at positions 46 and 108 of the fourth exon of NQO2. **(B)** Western blot of NQO2 in HCT116 (WT) and five knock out clones (numbered 1–5) transfected with the paired guide RNA. Four of the clones (2–5) had no detectable amount of NQO2. A non-specific band (*) was detected by the polyclonal anti-NQO2 antibody. **(C)** PCR amplification of exon 4 of NQO2 in HCT116 and HCT116 ΔNQO2 clones. Clone 1 showed a product size of 425 bp as expected, clones 2 and 4 did not have a PCR product, and clones 3 and 5 had a shorter PCR amplicon (top). PCR products were separated on a 3% polyacrylamide gel. Regions upstream and downstream of exon 4 were also individually amplified (bottom). A PCR product was observed for all clones in the 5′ flank and 3′ flank regions of exon 4. Thus, the two flanking regions of exon 4 exist in the genome but are disjointed in clone 2 and 4. PCR products of the flanking regions were separated on a 1% agarose gel.

*NQO2* was disrupted from HCT116 colon carcinoma cells using CRISPR guided Cas9 nickase to generate single stranded breaks at two neighboring sites in the fourth exonic region (Leung, 2015). The plasmid pSpCas9n(BB)-2A-Puro (PX462) encoding both the CRISPR guided RNA cassette and Cas9 nickase was a gift from Dr. Feng Zhang (Addgene plasmid # 48141). Using an online CRISPR Design Tool (http://crispr.mit.edu/), CRISPR motifs targeting nucleotide 46 and 108 of exon 4 were identified as the top sequences with minimal off-targets (Figure 1). Oligonucleotides corresponding to the pair of guide RNAs (sgRNA) were cloned into PX462 according to previous protocol (Ran et al., 2013) to produce the two vectors NQO24_46 and NQO24_108 (Supplementary Table S1). HCT116 cells at 70% confluence were then co-transfected with the sequence-verified vectors NQO24_46 and NQO24_108 using Lipofectamine 2000 (Life Technologies) according to the manufacturer’s directions. The media was supplemented with 0.7 μg/ml of puromycin (Alfa Aesar, Ward Hill, MA, United States) 24 h after transfection, and cells were grown in the presence of puromycin for 72 h. Surviving cells were trypsinized and serially diluted into 96-well plates supplemented with puromycin to select for single colonies carrying the transfected plasmid(s). After 2 weeks, five clones were isolated and expanded.

Cellular NQO2 was detected by Western blotting using rabbit anti-NQO2 polyclonal antibodies (a gift from Dr. Tim Haystead at Duke University). Cell lysates from each clone were resolved using SDS-PAGE, and then transferred onto a PVDF membrane. After blocking with 5% non-fat milk, the blot was probed for NQO2 was using a 1:1,000 dilution of the rabbit anti-NQO2 antibody and visualized with 1:5,000 fluorescently labeled secondary goat anti-rabbit antibody (Licor). To validate the excision of the targeted sequence on exon 4, genomic DNA was isolated from each of the HCT116^ΔNQO2^ clones and parental HCT116 cells using a commercial DNA extraction kit (Froggabio). Primers exon4_F1 and exon4_R1 were designed using Primer-Blast (http://www.ncbi.nlm.nih.gov/tools/primer-blast/) to amplify exon 4 of NQO2 (Supplementary Table S2). PCR was performed using Taq polymerase according to the manufacturer’s instructions (Biobasic). PCR products were resolved on a 3% polyacrylamide gel to detect the presence of an amplified DNA fragment from NQO2 exon 4. Since no PCR products were detected in clones 2 and 4, two additional primers (exon4_F2 and exon4_R2) were designed to amplify DNA regions that flanked the targeted cut sites (Supplementary Figure S1; Supplementary Table S2). PCR amplification of these flanking regions would determine whether the exon 4 was present but disjointed or was completely absent.

### Human Quinone Reductase 2 Expression Constructs

The protein sequences for NQO2 from Mallard duck (*Anas platyrhynchos*, accession XP_005022918), alligator (*Alligator mississippiensis*, accession XM_019490388), and Tibetan frog (*Nanorana parkeri*, accession XM_018564348) were used as representatives for extant bird, reptile, and amphibian NQO2s. The sequences were reverse-translated and codon optimized; KasI and BamH1 sequences were incorporated at the beginning and end of the protein coding sequences to facilitate downstream cloning. The gene sequences were synthesized by Integrated DNA Technologies (IDT, Coralville, Iowa); the sequences were verified by NGS (Illumina MiSeq) and supplied as a plasmid with a kanamycin marker. The coding sequences were cloned into pProEX-HTa (Invitrogen) using the KasI and BamH1 restriction sites, to yield constructs that would express the NQO2 genes as hexa-histidine tagged proteins, containing a TEV protease cleavage site. After TEV protease cleavage the proteins contain an extra glycine-alanine sequence at their N-terminus but are otherwise identical to the wild-type protein sequence.

### Protein Expression and Purification

NQO2 was expressed and purified following a previously published protocol (Leung et al., 2012). NQO2 constructs were maintained with ampicillin and expressed in *E. coli* BL21(DE3) in 4 L of the autoinduction ZYP-5052 media (Studier, 2005). The cells were grown overnight with shaking at 37°C to an OD_600_ of 7–8. After protein expression, cells were sedimented at 8,800 g for 15 min, resuspended in 50 mM sodium phosphate, 0.5 M NaCl, pH 7.5 (NTA-Buffer-A), flash-frozen in liquid nitrogen and stored at −80°C. For cell lysis, cells were thawed and treated with DNase and lysozyme, dispersed with a Dounce homogenizer, and lysed using a French tissue press. The lysed cell solution was supplemented with NTA-Buffer-B (50 mM sodium phosphate, 500 mM NaCl, 250 mM imidazole, pH 7.5) to yield a final imidazole concentration of 25 mM, and membranes were removed by centrifugation at 4°C and 100,000 × g for 1 h 20 min. The supernatant was loaded on Ni^2+^-affinity resin (IMAC Sepharose, Cytiva) and washed with 100 ml of NTA-Buffer-A with 25 mM imidazole, and eluted with NTA-Buffer-B. After this first purification step, NQO2 was dialyzed against 100 mM Tris-HCl, 0.5 M NaCl, 5 mM EDTA, pH 8.2. The histidine affinity tag was completely cleaved off by incubation with Tobacco Etch Virus (TEV) protease in the presence of 5 mM DTT. NQO2 (pI ≈ 5.1) was applied to 1.6 × 15-cm Q-Sepharose HP (Cytiva) anion exchange column equilibrated with 20 mM Tris, 1 mM EDTA at pH 8.4. NQO2 was eluted with a salt gradient from 0 to 500 mM. Finally, NQO2-containing fractions were pooled and concentrated in preparation for gel filtration chromatography. To reconstitute fully with FAD, the concentrated protein samples were kept on ice and treated with 3 M guanidine HCl, and 10 mM FAD, zinc chloride and DTT. After gentle mixing and incubation on ice, the protein samples were applied to 2.6 × 65-cm column of Superdex-200 preparatory grade resin (Cytiva), previously equilibrated with 50 mM Tris, 150 mM NaCl, 10 µM FAD, 10 µM Zn^2+^ at pH 8.0. After gel filtration, NQO2 was dialyzed against 50 mM HEPES, 100 mM NaCl, pH 7.4, concentrated with Amico Ultra-centrifugal filter units, flash frozen in liquid nitrogen, and stored at −80°C.

### Steady-State Kinetic Analysis

Steady-state kinetic analysis of NQO2 homologs was carried out with BNAH (5–340 µM) and menadione (2–100 µM) with human NQO2 at 0.46 nM, reptile NQO2 at 2.06 nM and bird NQO2 at 4.89 nM. Stock solutions of BNAH and menadione were made fresh in methanol. Reaction progress was monitored by following BNAH oxidation at 355 nm in 100 mM HEPES, pH 7.4 at 25°C in a 1 ml volume with Cary 100 spectrophotometer. Each reaction was repeated in triplicate or greater. The initial rates were globally fit to a ping-pong bi-substrate Michaelis Menten equation (Eq. 1, below) to obtain *K*_M_ for both substrates and *K*_I_ for menadione using Prism (www.graphpad.com). Steady-state kinetic analysis with conventional dihydronicotinamide cosubstrates was carried out using NADH and NADPH at 40 μM to 12 mM concentrations, and menadione at 20 µM or 40 μM, with human NQO2 at 6–24 μM, reptile NQO2 at 5–10 μM, and bird NQO2 at 10–20 µM. Reaction progress was monitored by following the oxidation of NAD(P)H by its decrease in absorbance at 340 nm (*ε* = 6220 M^−1^ cm^−1^) in 100 mM Tris-HCl, pH 8, at 25°C in a 96-well plate format using a Synergy H1 plate reader for 1 h 30 min, with pathlength correction and an absorbance reading every minute. Each reaction was repeated in triplicate or greater.

$$rate=\frac{k_{cat}\left[NADH\right]\left[menadione\right]}{K_{NADH}\left[menadione\right]\left(1+\frac{\left[menadione\right]}{K_{I,menadione}}\right)+K_{menadione}\left[NADH\right]+\left[NADH\right]\left[menadione\right]}$$ (1)

### Phylogenetic Analysis

NQO1 and NQO2 amino acid sequences and bacterial NQO3 (MdaB) were collected from the NCBI database. After an initial alignment by MAFFT, sequences with unusual lengths which may be incomplete were removed to give a total of 172 unique NQO1, NQO2 and NQO3. Phylogenetic tree construction was done using PhyloBot (Hanson-Smith and Johnson, 2016). The phylogenetic tree was further confirmed by MEGA X where the optimum evolutionary model was first determined to best model the substitution pattern. 57 evolutionary models were evaluated and ordered by Bayesian Information Criterion (BIC) score. The model with the lowest BIC score, JTT with (+G) 1 rate categories was then used to construct a phylogenetic tree by maximum likelihood with 100 bootstrap replications for testing the phylogeny in MEGA X. The test inference was a nearest-neighbour-interchange tree.

### Cell Culture and Analysis

HCT116 cells were cultured in McCoy’s 5A (modified) medium with glutamine. Cell culture media was supplemented with 100 units/mL penicillin and 0.1 mg/ml streptomycin. To assess the ability of NQO2 to catalyze redox reactions in cells, activation of CB1954 by NRH was used as a reporter of NQO2 activity (Nolan et al., 2012). HCT116 or HCT116ΔNQO2 cells were seeded at 1,000 cells per well in 96-well plates and were allowed to attach overnight. Cells were then treated with: 1) 7.8–1,000 μM of CB1954 and 6–200 μM of NRH, 2) 7.8–1,000 μM of CB1954 alone, 3) 4.8–625 μM of NRH alone or 4) nothing (*n* = 3). Cells were allowed to grow for an additional 48 h before they were counted using sulforhodamine B (SRB) assay, which stains for total protein content of fixed cells (Skehan et al., 1990). Cells were first fixed with trichloroacetic acid (TCA) for 1 h at 4°C, then washed with water three times. They were then stained with SRB dye (Sigma) for 20 min at room temperature and washed with 1% acetic acid. The dye was re-solubilized in 10 mM Tris base and its absorbance was measured using a multi-reader at 560 nm (Victor multi-plate reader, Perkin Elmer). Absorbance readings of treated cells were normalized against untreated cells, and the data were fitted to a dose-response curve and graphed using Prism (GraphPad Software Inc.).

## Results

### Human Quinone Reductase 2 Requires Exogenous Dihydronicotinamide Cosubstrates for Enzymatic Function

NQO2 is unusual because related quinone reductases use NADH and/or NADPH as reducing cosubstrates, whereas NQO2 uses these cosubstrates extremely inefficiently. This raises the question of whether NQO2 can catalyze reduction of electrophiles in cells using an endogenous reducing cosubstrate. In fact, NQO2 has been demonstrated to catalyze the reduction of an electrophile in cells, but only when exogenous dihydronicotinamide cosubstrates are provided. Specifically, the ability of NQO2 to function as an enzyme in cells was demonstrated using the anticancer prodrug CB 1954 (5-(aziridin-1-yl)-2,4-dinitrobenzamide). When reduced with 4 electrons, CB1954 becomes a bifunctional alkylating agent (2,3-dinitrobenzamide) that is much more toxic than the prodrug (Knox et al., 2000). In human cells, the transformation of CB1954 to a bifunctional alkylating agent is catalyzed by NQO2. Knox and co-workers demonstrated this using Chinese hamster V79 cells that have a low level of NQO2 activity and are relatively resistant to CB 1954. However, when these cells were transfected with an NQO2-expressing vector, their sensitivity to CB1954 increased approximately 100-fold, but only in the presence of exogenous dihydronicotinamide riboside (NRH) or other small dihydronicotinamide compounds (Knox et al., 2000). The conclusion from this work was that an endogenous cosubstrate for NQO2 was not present in these cells.

To further validate the importance of NQO2 for reduction of CB1954 and the requirement for exogenous dihydronicotinamide cosubstrates to enable NQO2 catalytic activity, we created human NQO2-knockout cell lines. The NQO2 gene was disrupted in HCT116 colon carcinoma cells using a CRISPR-Cas9 dual nickase system that targeted NQO2 exon 4. This work yielded two NQO2-knockout HCT116 cell lines that have been used to probe the cellular function of NQO2. NQO2 is absent from these cell lines, as assessed by genome analysis and western blotting (Figure 1).

**FIGURE 2:**
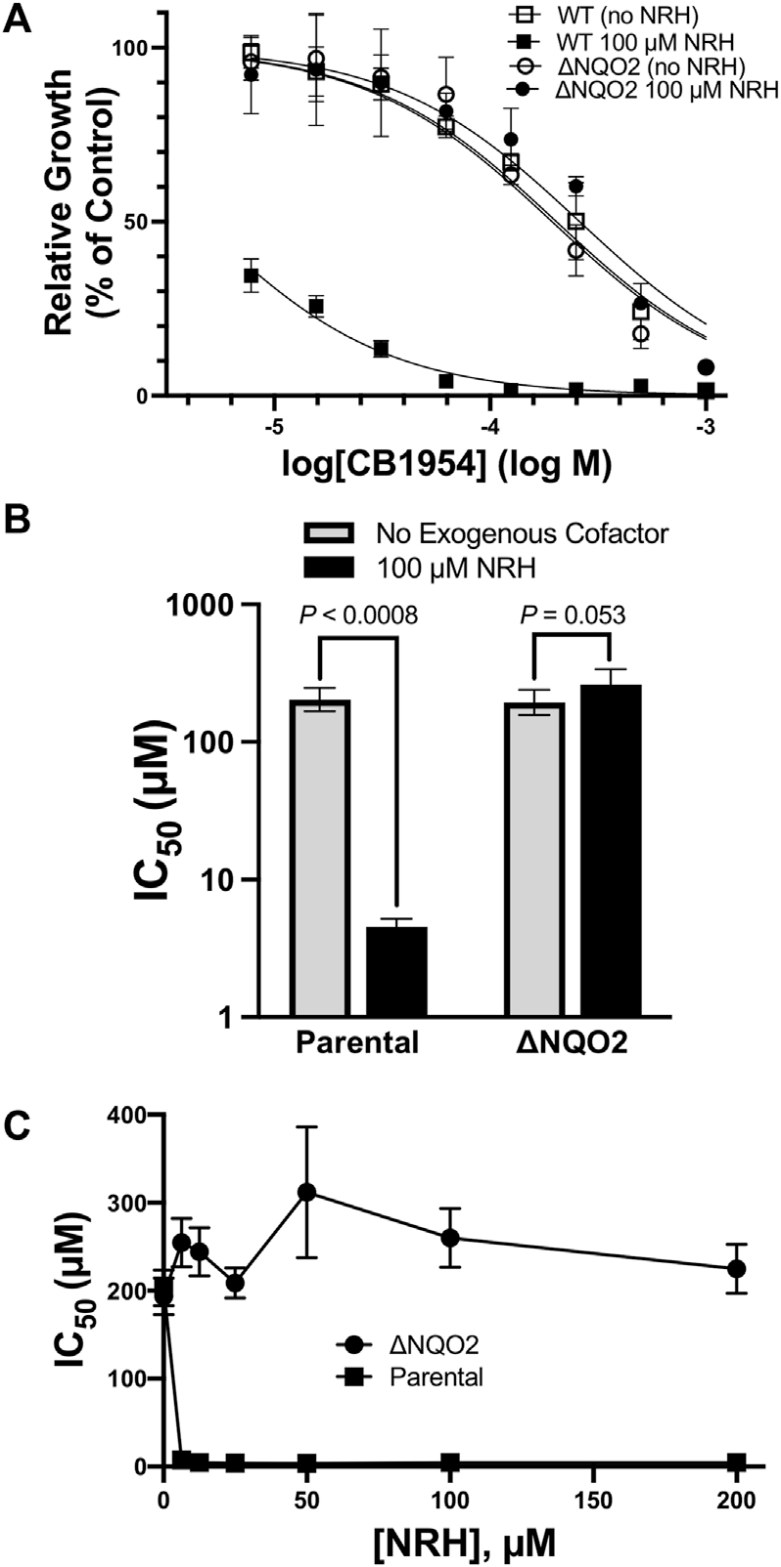
Effect of NQO2 Knockout on CB1954 Toxicity. **(A)** HCT116 parental (squares) or NQO2 knockouts (ΔNQO2, circles) were seeded and then grown in varying concentrations of CB1954 in the absence (empty markers) or presence (filled markers) of 100 µM dihydronicotinamide riboside (NRH). Cell growth was measured by SRB assay and normalized to untreated cells; measurements were made in triplicate and the error bars represent 95% confidence intervals. **(B)** IC_50_ values in both the absence and presence of 100 µM NRH values cells were derived from the dose-response curves fitted to the data in **(A)**. The error bars represent 95% confidence intervals; two-tailed t-tests were used to calculate *P* values for the null hypothesis, namely that the IC_50_ values are not significantly different. **(C)** Sensitivity of HCT116 parental (squares) and ΔNQO2 (circles) cells to varying concentrations (0, 6, 12.5, 25, 20, 50, 100, and 200 µM) of NRH. Cells were treated as in **(A)** but with varying NRH concentrations to obtain dose-response curves and IC_50_ values. The error bars represent 95% confidence intervals. Two-tailed t-tests between the IC_50_ for parental cells with no NRH and all of the concentrations of NRH indicate a significant decrease in IC_50_, with *p* values consistently less than 0.0008; in contrast, the effect of NRH on the NQO2 knockouts appears to result in a slight increase in IC_50_, although the P values are less significant, ranging from *p* ≈ 0.05 (at 6.25, 50, and 100 µM NRH) to *p* = 0.11, 0.58, and 0.41 at 12.5, 25, and 100 µM NRH.

To assess the effects of CB1954 on cells, media was supplemented with various concentrations of CB1954, cells were grown for 48 h, and cell density was assayed and compared to untreated cells. HCT116 cells had IC_50_ values of approximately 200 µM for CB1954, and this IC_50_ value did not increase when the gene for NQO2 was disrupted (Figures 2A,B). The lack of an effect of removing NQO2 from cells demonstrates that NQO2 has no significant ability to catalyze reduction of CB1954 using endogenous cellular cosubstrates or reducing agents. However, when 100 µM NRH was added to the cell culture media, the IC_50_ of CB1954 dropped 45-fold for parental HCT116 cells, but remained unchanged in the case of the NQO2-knockout cells (Figures 2A,B). Note that in the absence of CB1954, 100 µM NRH had no significant effect on cell growth. HCT116 cells were further tested with a range of exogenous NRH concentrations, and it was found that a concentration of 5–10 µM exogenous NRH was sufficient to fully lower the IC50 of CB1954 (Figure 2C). Together, these results demonstrate that NQO2 has no significant capacity to reduce CB1954 in cells in the absence of an exogenous cosubstrate, and only relatively small concentrations of exogenous NRH are required for more robust NQO2-mediated catalysis.

### Cosubstrate Specificity of Human Quinone Reductase 2

Previous steady-state kinetics studies compared the enzymatic function of NQO2 using NADH and small dihydronicotinamide cosubstrates such as NRH, NMeH (*N-*methyl-1,4-dihydronicotinamide), and SCDP (1-(3-sulfonatopropyl)-1,4-dihydronicotinamide. BNAH (1-benzyl-1,4-dihydronicotinamide) is another small dihydronicotinamide cosubstrate, and steady-state kinetics demonstrates that NQO2 uses BNAH with a catalytic efficiency that approaches the diffusion-controlled limit of approximately 10^8^ M^−1^•s^−1^ (Table 1). NRH, NMeH, SCDP, and BNAH are all similar in that they have a small group (ribosyl, methyl, sulfylpropyl, or benzyl) attached to the pyridine nitrogen of the nicotinamide, which contrasts with NADH that has ADP-ribosyl attached to the nicotinamide. NADH is a much poorer cosubstrate for NQO2 and in our experience the activity of human NQO2 with NADH is difficult to measure, requiring extended incubations with relatively high concentrations of enzyme. The kinetic parameters of NQO2 with NADH as a cosubstrate include *k*_CAT_ values of 1 s^−1^ or less, *K*_M_ values around 0.5 mM and catalytic efficiencies that are 100 to 10,000-fold less than those observed with the smaller cosubstrates (Table 1). It is noteworthy that the closely related quinone reductase, human NQO1, which has well-defined cellular roles in the catalytic reduction of quinones and other electrophiles, exhibits a *k*_cat_ of 240 ± 12 s^−1^ and a *K*_M_ for NADH of 290 ± 48 µM in its reaction with menadione, for an overall catalytic efficiency of 8.3•10^5^ M^−1^•s^−1^ (Al Massri, 2017); these values for NQO1 obtained in our lab compare favourably to those measured previously, for example *k*_cat_ of 515 ± 50 s^−1^ and *K*_M_ for NADH of 70 ± 8 µM to yield an overall efficiency of 7.4•10^6^ M^−1^•s^−1^ (Wu et al., 1997). In addition, NQO1 is able to use NADPH even more efficiently than NADH (Tedeschi et al., 1995) and NADPH is generally thought to be in greater concentration in cells than NADH (Veech et al., 1969). On this basis, NQO1 and NQO2 have followed very different evolutionary trajectories in terms of their abilities to use the common cellular reducing cosubstrates NADH and NADPH.

**TABLE 1:** Steady-state kinetic parameters for cosubstrate use by human NQO2.

Cosubstrate	*k*_cat_ (s^−1^)	*K*_M_ (µM)	*k*_cat_/*K*_M_ (M^−1^• s^−1^)	Acceptor	References
NADH	2.6	252	1,000	Menadione	(Wu et al., 1997)
NADH	0.6	330	2,000	DCIP	(Chen et al., 2000)
NADH	0.06 ± 0.008	1,090 ± 440	55	Menadione	(Al Massri, 2017)
NRH	45 ± 5	28 ± 2	1.5–1.6 • 10^6^	DCIP	(Chen et al., 2000)
SCDP	981 ± 90	142 ± 23	6.9 • 10^6^	Menadione	(Leung and Shilton, 2015)
NMeH	462 ± 103	18 ± 3	2.6 • 10^7^	Menadione	(Kwiek et al., 2004)
BNAH	1,190 ± 170	96 ± 8	1.2 • 10^7^	Menadione	this work

### Evolution of NQO1 and NQO2

NQO1 and NQO2 are closely related in sequence and structure but evolved to have very different cosubstrate specificities. NQO1 is an enzyme regulated as part of the Keap1-Nrf pathway that responds to oxidative and xenobiotic stress (Sihvola and Levonen, 2017). NQO1 has a C-terminal domain that is not present in NQO2, while NQO2 has a metal binding site and an unusual cosubstrate specificity that is difficult to reconcile with an enzymatic function in cells. The evolution of quinone reductases was analyzed in 2006 by Vasiliou, Ross and Nebert (Vasiliou et al., 2006) and since that time many more animal genomes have been sequenced, facilitating a more comprehensive analysis.

**FIGURE 3:**
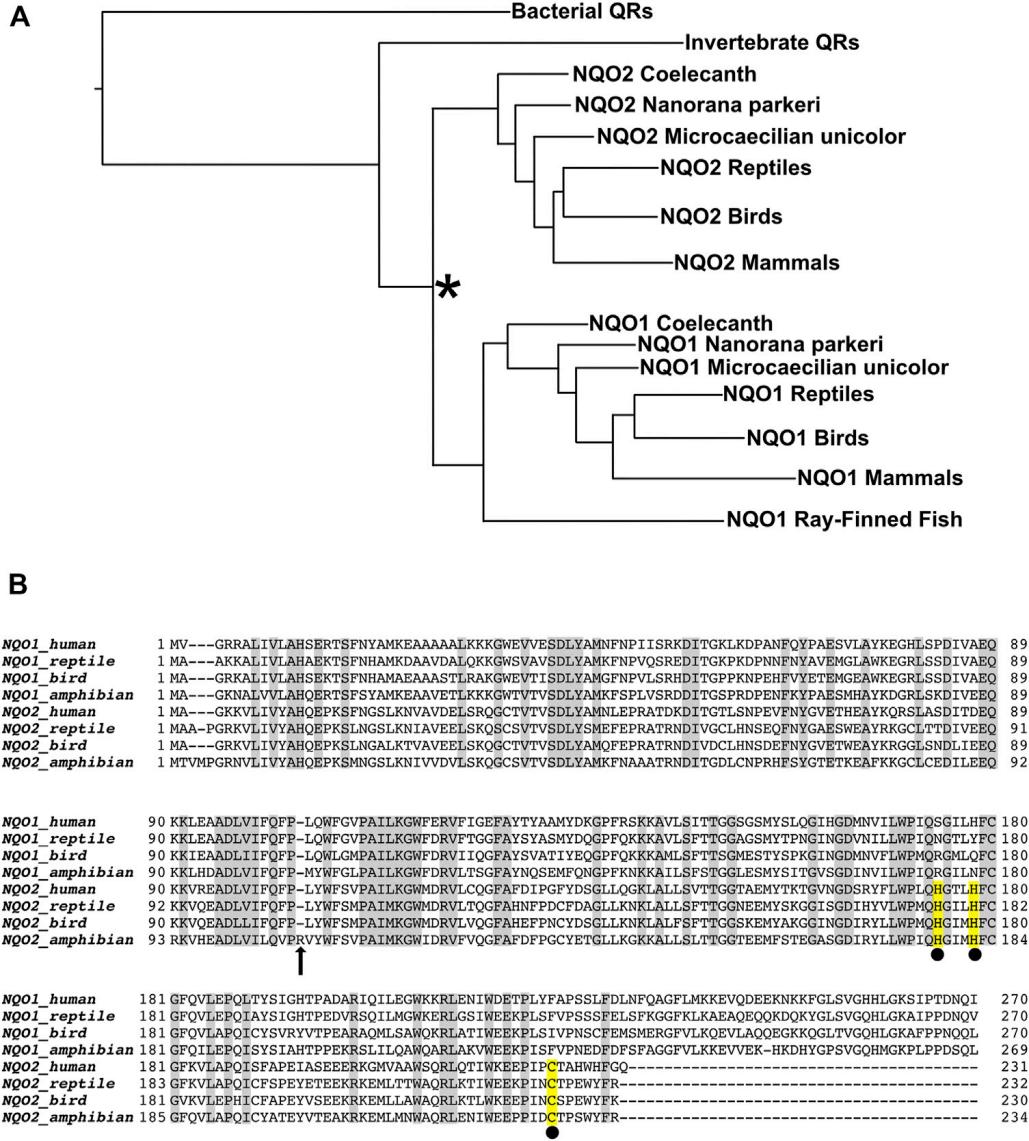
Evolution of Quinone Reductases. **(A)** Simplified phylogenetic tree calculated from 172 unique quinone reductase sequences. Duplication of an NQO gene (indicated by the asterisk) occurred in an ancient vertebrate fish (*Euteleostomi*) to produce the NQO1 and NQO2 enzyme families. The *Euteleostomi* then diverged into subgroups *Actinopterygii*, which includes extant ray-finned fishes, and the *Sarcopterygii* that includes the tetrapods. NQO1 is present in all vertebrates, whereas NQO2 was apparently lost in the ray-finned fishes, which have only the NQO1 gene. NQO2 is present in the lobe-finned fish, *Latimeria chalumnae* (coelacanth) and some amphibian genomes, including the High Himalayan frog, *Nanorana parkeri,* and *Microcaecilia unicolor*. NQO2 is also present in all the amniotes, namely the reptiles, birds, and mammals. **(B)** Sequence alignment of extant NQO1 and NQO2 enzymes from human and representative reptile (*Alligator mississippiensis*), bird (*Anas platyrhynchos*) and amphibian (*Nanorana parkeri*). The most prominent differences between the enzymes are the conserved C-terminal extension of NQO1, and the conserved metal binding site of NQO2, indicated by the yellow highlighted residues with black spheres underneath. Both the reptile and bird NQO2 sequences have been expressed in *E. coli*, purified, and characterized, whereas NQO2 from *Nanorana parkeri* could not be expressed as a recombinant protein despite several attempts. We believe the enzyme from *N. parkeri* is no longer functional due to residue changes, including insertion of arginine (indicated by the arrow at position 107) in a highly conserved region that interacts directly with the isoalloxazine of FAD.

Sequences of NQO1 and NQO2 from extant metazoans were aligned, along with archaeal NQO5 genes as the out-group, to generate a phylogenetic tree using the PhyloBOT web portal (Hanson-Smith and Johnson, 2016). A summary version of the phylogenetic tree is presented in Figure 3A. The phylogenetic tree indicates an ancestral quinone reductase at the root of the tree that evolved to yield the quinone reductase (QR) enzymes present in extant bacteria and archaea, and QR enzymes in animals. In the case of the vertebrates, a duplication of an ancestral QR gene took place, giving rise to the NQO1 and NQO2 families. However, these two QR genes are not distributed uniformly within vertebrates. NQO1 is present and conserved in the ray-finned fishes, lobe-finned fishes, amphibians, and amniotes (reptiles, birds, and mammals). In contrast, we could not find an NQO2 gene present in any of the extant ray-finned fishes. NQO2 genes were present in all amniotes and in some, but not all, of the 18 available amphibian genomes, as well as the lobe-finned fish, *Latimeria chalumnae* (i.e., West Indian Ocean coelacanth). Extant amphibians and lobe-finned fishes are thought to be related to ancient organisms that were on the evolutionary pathway to the first land-dwelling animals, which ultimately became the amniotes. Since NQO1 in the ray-finned fishes is conserved with NQO1 in lobe-finned fishes, amphibians, and amniotes, the QR gene duplication giving rise to NQO1 and NQO2 must have taken place before the bony fish separated into the lobe-finned (*sarcopterygii*) and ray-finned (*actinopterygii*) fishes, approximately 450 MYA (Hedges, 2009). In the case of the ray-finned fishes, we have not found a recognizable NQO2 enzyme in the available genomes, indicating that the gene was lost at an early state in their evolution.

NQO2 is present to varying degrees in the available amphibian genomes. There are currently 18 sequenced amphibian genomes of the anuran and caecilian orders in the NCBI database. A comprehensive search for an NQO2 gene was done using all 18 of the available sequenced amphibian genomes. The three sequenced caecilian genomes of *Geotrypetes seraphini*, *Rhinatrema bivittatum*, and *Microcaecilia unicolor* have both NQO1 and NQO2 genes. Among the frogs, some of them such as *Xenopus* have lost NQO2. A series of tBLASTn searches against individual amphibian genomes with NQO2 gene of *M. unicolor* and NQO1 gene of *X. tropicalis* revealed that frogs such as *Nanorana parkeri*, *Pyxicephalus adspersus*, *Leptobrachium leishanense*, *Limnodynastes dumerilii*, *Oophaga pumilio*, *Rana temporaria*, and *Scaphiopus couchii* have two distinct NQO genes resembling NQO1 and NQO2. However, it seems likely that some of the NQO2 genes found in the amphibians are not functional due to loss or scrambling of exons and/or significant changes in conserved regions of the protein. The inconsistent presence of NQO2 in the amphibians and its absence in ray-finned fishes suggests that its function in the land-dwelling amniotes is not as advantageous for semi-aquatic or aquatic organisms.

The evolutionary origins and trajectories of NQO1 and NQO2 tell us something about the major differences between extant enzymes. The structure of recombinant NQO2 exhibited a zinc binding site (Foster et al., 1999), but the metal is copper when the enzyme is purified from human red blood cells (Kwiek et al., 2004). The metal is coordinated in a distorted tetrahedral geometry by two histidines, H174 and H178, combined with the thiol and main-chain carbonyl of a cysteine, C223, consistent with a Type I copper binding site (Koch et al., 1997). Although the function of the NQO2 copper binding site is not known, it is not present in NQO1 and represents a major difference between the two proteins (Figure 3B). Another major difference between the enzymes is the extended C-terminal domain of NQO1. The 43 residue C-terminal domain is an important structural feature that sets NQO1 apart from NQO2 and related quinone reductases such as the bacterial NQO3. The function of the NQO1 C-terminal domain is not known.

### Properties of Extant Human Quinone Reductase 2 Enzymes

**FIGURE 4:**
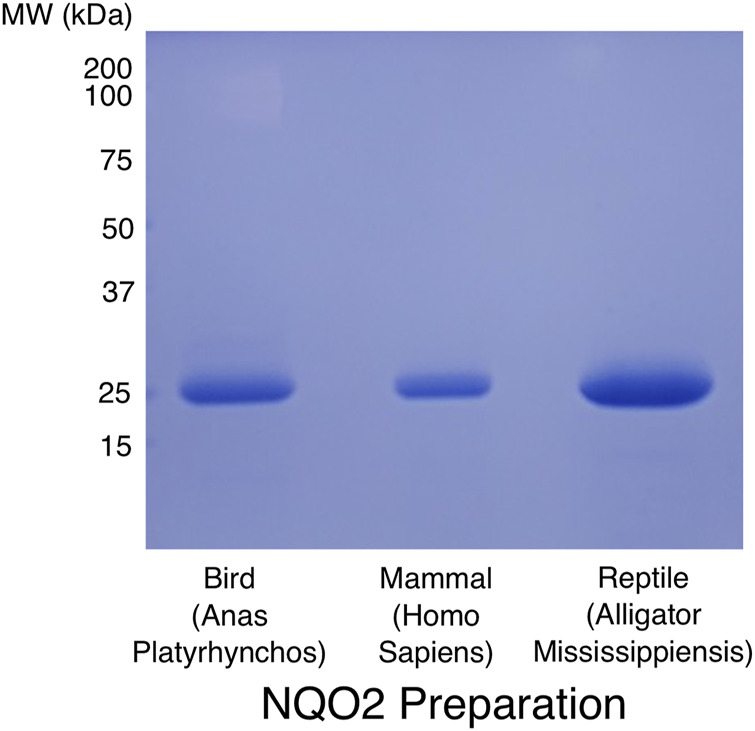
SDS-PAGE analysis of purified NQO2 preparations. The final preparations of NQO2 enzymes used for kinetic analyses were run on a 12% SDS-PAGE gel and stained with Coomassie brilliant blue R250. Codon-optimized coding sequences for NQO2 from *Anas Platyrhynchos* (predicted MR 26.7 kDa) and *Alligator Mississippiensis* (predicted MR 27.7 kDa) were expressed and purified using the same protocol developed for the human enzyme (predicted MR 26.0 kDa; Leung et al., 2012). The proteins were expressed as hexa-histidine tagged constructs and purified using Ni^2+^-affinity chromatography, followed by removal of the affinity tag using TEV protease, anion exchange chromatography, and gel filtration chromatography.

The evolutionary analysis of NQO1 and NQO2 indicates they diverged in at least two important ways early in the evolution of amniotes, namely in the generation of the conserved C-terminal domain in NQO1 and the metal binding site in NQO2. From a sequence and structural perspective, the determinants for cosubstrate specificity are not obvious, and in fact it is not clear whether the unusual cosubstrate specificity of NQO2 is conserved among the amniotes. To address this question, we cloned and expressed NQO2 enzymes from mallard duck (*A. platyrhynchos*), alligator (*A. mississippiensis*), and Tibetan frog (*N. parkeri*). The three enzyme sequences were codon optimized for expression in *E. coli* and synthetic DNA coding sequences were cloned into the same expression vector we use for human NQO2. Protein expression and purification followed an identical protocol developed for the human enzyme (Leung et al., 2012). The final preparations for NQO2 from *A. platyrhynchos, Homo sapiens*, and *A. Mississippiensis* are shown in Figure 4.

Recombinant NQO2 from *N. parkeri* was not expressed in *E. coli*, despite several attempts. A closer look at the sequence reveals a single residue insertion after residue 106 (residue 103 in human NQO2, Figure 3B). This insertion occurs in a highly conserved region of the protein, and in fact this sequence interacts directly with the isoalloxazine of FAD. On this basis, we believe that the protein is not able to bind FAD and/or fold properly. This mutation and the instability of the *N. parkeri* NQO2, combined with genetic changes and rearrangements in other amphibian NQO2s, and the total absence of NQO2 in some amphibians, is consistent with the idea that NQO2 does not provide a strong selective advantage to the amphibians.

The reptile and bird NQO2 enzymes gave high yields of soluble and functional recombinant NQO2 and were analyzed using steady-state kinetics to determine their cosubstrate specificity (Tables 2, 3). From these results, the reptile and bird NQO2 appear similar to the human enzyme in terms of their ability to efficiently catalyze quinone reduction using a small dihydronicotinamide cosubstrate, BNAH. The reactions of the NQO2 enzymes with NADH and NADPH were observable but very slow. To obtain kinetic parameters for reaction rate and Michaelis constant for NAD(P)H, the assays were carried out in a microplate reader over a relatively long time and using high concentrations of NADH or NADPH. With the use of small volumes and high enzyme and cosubstrate concentrations, detectable changes in reduced cosubstrate concentration were observed. The values are provided in Table 3 and the extremely inefficient use of NADH and NADPH in the human enzyme is conserved in both the reptile and bird enzymes. The inability of NQO2 to efficiently use these cosubstrates is therefore conserved among the amniotes.

**TABLE 2:** Steady-state kinetic constants for NQO2 with BNAH as cosubstrate.

Species	*k*_cat_ (s^−1^)^a^	*K*_M, BNAH_ (µM)^a^	*K*_M, menadione_ (µM)^a^	*K*_I, menadione_ (µM)^a^	*k*_cat_/*K*_M, BNAH_ (M^−1^•s^−1^)
*Homo Sapiens*	1,190 ± 72	96 ± 8	65 ± 7	ND^b^	1.2 • 10^7^
*Alligator Mississippiensis*	173 ± 30	35 ± 17	36 ± 11	24 ± 19	0.49 • 10^7^
*Anas Platyrhynchos*	128 ± 11	6.8 ± 4.5	3.5 ± 1.6	11 ± 11	1.9 • 10^7^

a Indicated errors are the approximate 95% confidence limits.

b There was no observed substrate inhibition at the concentrations of menadione used.

**TABLE 3:** Steady-state kinetic parameters for NQO2s with NADH and NADPH.

Species	*k*_cat_ (NADH) (s^−1^)^a^	*K*_NADH_ (µM)^a^	*k*_cat_/*K*_NADH_ (M^−1^•s^−1^)	*k*_cat_ (NADPH) (s^−1^)^c^	*K*_NADPH_ (µM)^c^	*k*_cat_/*K*_NADPH_ (M^−1^•s^−1^)
^b^ *Homo Sapiens*	0.0792 ± 0.018	26,800 ± 10,000	3.0 ± 1.8	^c^0.098	^c^42,600	^c^2.3
^b^ *Alligator Mississippiensis*	0.116 ± 0.025	8,000 ± 3,000	15 ± 8	0.019 ± 0.003	700 ± 300	2.7 ± 1.6
^b^ *Anas Platyrhynchos*	0.0206 ± 0.005	1,600 ± 700	13 ± 9	ND	ND	^c^1.8

a Indicated errors are the approximate 95% confidence limits.

b For non-linear fitting to the rate equations, the values for *K*_menadione_ and *K*_I, menadione_ were fixed at the values determined with BNAH as cosubstrate, in Table 2.

c For these measurements, NQO2 could not be saturated with cosubstrate, and the lack of a plateau in rate at high concentrations of cosubstrate made estimation of rate parameters and associated errors particularly difficult.

## Discussion

NQO2 is a fascinating enzyme precisely because it has evolved to become a very *inefficient* catalyst with the commonly available cellular cosubstrates, NADH and NADPH. Compared to its much more efficient paralogue, NQO1, the catalytic efficiency for NQO2 is lower by a million-fold or more. It may be that there exist other reducing cosubstrates in the cell, but they do not seem to be sufficiently abundant for sustained catalysis. Our experiments with HCT116 NQO2 knockouts demonstrate that the reduction and activation of the CB1954 toxin by NQO2 is completely dependent on the addition of an exogenous dihydronicotinamide cosubstrate, such as NRH.

We have also shown that the unusual kinetic properties of mammalian NQO2 are conserved in the American alligator and mallard duck, two species chosen to represent the other major groups of amniotes, the reptiles, and birds. Tetrapods evolved initially from an amphibian species, and then diverged into two separate lineages early in amniote evolution, with the synapsida branch leading to the mammals, and reptilia giving rise to reptiles and birds (Ford and Benson, 2020). Therefore, despite the very early divergence between the mammals and reptiles/birds, the defining properties of NQO2, namely the metal binding site and unusual cosubstrate specificity, are conserved in several extant mammals, a bird, and a reptile. On this basis, we believe that the unusual cosubstrate specificity is almost certainly conserved among all amniotes. Therefore, after a gene duplication event involving a common ancestor of NQO1 and NQO2, the two enzymes diverged in function. NQO1 evolved as a *bona fide* detoxification enzyme, regulated as part of the Keap1-Nrf2 pathway and able to efficiently use NAD(P)H for reduction of potentially harmful quinones and other electrophiles. In contrast, NQO2 evolved for other functions that apparently do not require a high level of catalytic efficiency. It is noteworthy that this evolution of NQO2 took place alongside NQO1, but only in amniotes. Amniotes differ from fish and amphibians in that the amniotic membrane surrounding the egg allowed these organisms to move from an aquatic to terrestrial existence. On this basis, the functional divergence of NQO2 would have occurred roughly 350 MYA, as the tetrapods were evolving. NQO2 is not present in extant ray-finned fish, indicating that for these organisms that maintained an aquatic existence, NQO2 was lost from the genome, allowing NQO1 to evolve independently. For the amphibians, some species, such as *Xenopus laevis*, have lost NQO2, while other species have retained NQO2, although in many cases it is not clear that the enzyme is functional. In the case of the High *Himalaya frog*, *N. parkeri*, the NQO2 gene contains an insertion that results in the addition of an arginine residue in a highly conserved region of the protein that interacts directly with the isoalloxazine of FAD. We were unable to express this protein in *E. coli*, probably because the insertion disrupts folding and/or binding of the FAD prosthetic group. The evolutionary trajectory of NQO2 indicates that its initial function was apparently critical for a terrestrial existence, but dispensable for an aquatic or semi-aquatic existence.

In terms of evolution, the question arises as to whether the extremely inefficient use of NAD(P)H by extant NQO2 enzymes resulted from passive genetic drift or whether it appeared through a process of natural selection. The observation that the inefficient use of common NAD(P)H cosubstrates is conserved throughout the amniotes is consistent with this property arising through natural selection; in other words, whatever function NQO2 has, its inefficient use of NAD(P)H confers a selective advantage. Since this property greatly limits the ability of NQO2 to function as an enzyme in cells, it suggests that the primary function of NQO2 is non-catalytic. At the same time, NQO2 has retained its ability to bind the FAD prosthetic group. On this basis, NQO2 may function in cell signaling as a flavin redox switch (Becker et al., 2011). It has previously been observed that reduction of the flavin cosubstrate and binding of chloroquine resulted in a change in conformation or dynamics, which could affect interactions of NQO2 with other molecules (Leung and Shilton, 2013). A recent manuscript implicating NQO2 in regulation of autophagy concluded that the mechanism may not require catalytic activity, but instead depend on signaling through ligand binding and changes in protein-protein interactions (Janda et al., 2021). Another possibility is that NQO2 signals through the production of reactive oxygen species, which have been detected *in vitro* (Reybier et al., 2011) and in cells (Cassagnes et al., 2015) during the reduction of quinones by NQO2 using a suitable cosubstrate such as NRH or BNAH. NQO1 functions as an efficient enzyme, but also has non-enzymatic functions that include regulating the degradation of intrinsically disordered proteins (IDPs) such as p53 by the 20S proteosome (Tsvetkov et al., 2010; Moscovitz et al., 2012). The diverse functions of NQO1 have been recently reviewed (Ross and Siegel, 2021), and with these non-enzymatic functions of NQO1 in mind, it is reasonable to think that NQO2 could have complementary non-enzymatic functions. On the other hand, it is still not clear why NQO2 has lost its catalytic ability with NAD(P)H as cosubstrate, while NQO1 has retained its ability to efficiently catalyze redox reactions using NAD(P)H.

Finally, given their very similar structures, the molecular mechanism of the differences in cosubstrate specificity between NQO1 and NQO2 is not obvious. In a previous study a chimeric NQO2 was created that included the C-terminal extension of NQO1, but the chimeric protein retained its preference for NRH over NAD(P)H, indicating that the C-terminal extension did not determine the cosubstrate specificity (Wu et al., 1997). Further analysis of the changes along the evolutionary pathways to extant NQO1 and NQO2 should help to illuminate the origins of the unusual cosubstrate preference of NQO2.

## Data Availability

The original contributions presented in the study are included in the article/Supplementary Material, further inquiries can be directed to the corresponding author.
