# Supplementary material for: The Unusual Cosubstrate Specificity of NQO2: Conservation Throughout the Amniotes and Implications for Cellular Function
Source: Front Pharmacol. 2022 Apr 20;13:838500. doi: 10.3389/fphar.2022.838500 (PMC9065289; doi:10.3389/fphar.2022.838500)
Supplement: Supplementary file 1 [file DataSheet1.PDF]

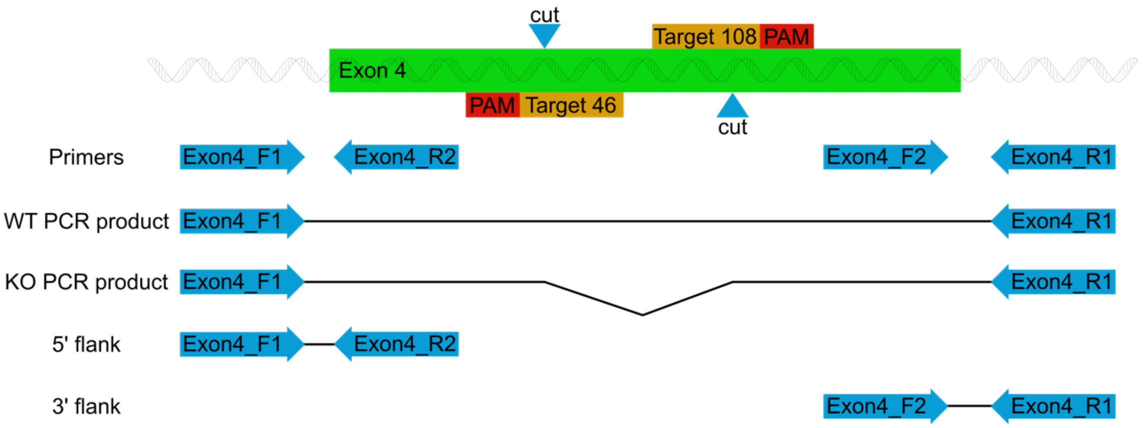

Figure S1 Cartoon Illustration of Primers Used for PCR Validation in of HCT116$^{\Delta NQO2}$ Cells.
PCR amplification of wild-type NQO2 gene using primers Exon4_F1 and Exon4_R1 will generate a PCR product of 425bp, while the PCR product of the knockout gene is estimated to be 363bp if exonuclease excise single stranded nucleotides up to the targeted cut sites. PCR amplification of the 5' flank and 3' flank regions in genomes where full-length PCR products are absent will show that the two ends of exon4 still exist in the genomic DNA. This suggests that a chromosomal translocation event has occurred.

Table S1 Oligonucleotides Used for CRISPR Cassette Construct.

Guide RNA oligos	Sequence
Exon4_46_top	CACCGACCTTTGCTTGTAGGCTTCG
Exon4_46_bottom	AAACCGAAGCCTACAAGCAAAGGT
Exon4_108_top	CACCGTGAGCAGAAAAAGGTTCGGG
Exon4_108_bottom	AAACCCCGAACCTTTTTCTGCTCA

Table S2 Oligonucleotides Used for PCR Validation of HCT116$^{\Delta NQO2}$ cells

Primers	Sequence
Exon4_F1	TGCTAGGTAGCAAGTGCTCAATC
Exon4_R1	CTTCCAGAAGCAGCACAAAACTC
Exon4_F2	CTGACCTAGTGATATTTCAGGTTTGTTTTTCTC
Exon4_R2	GTTTCCACTCCATAATTGAAAACCTCAGG
46oF1	ATGGAAGCAGGAACTCAAACTCA
46oR1	GTACATGCTGGCCTAGAAAACAC
108oF1	AGTAGGACATCACAGAAGTTGGG
108oR1	TTCTGCTCAAAGGTTTCGCC
